# Longitudinal risk of serious infections in patients with inflammatory arthritis on immunomodulating therapy compared to controls

**DOI:** 10.1093/rap/rkaf017

**Published:** 2025-02-12

**Authors:** Ingrid Egeland Christensen, Siri Lillegraven, Joseph Sexton, Tore K Kvien, Till Uhlig, Sella Aarrestad Provan

**Affiliations:** Center for Treatment of Rheumatic and Musculoskeletal Diseases (REMEDY), Diakonhjemmet Hospital, Oslo, Norway; Institute of Clinical Medicine, University of Oslo, Oslo, Norway; Center for Treatment of Rheumatic and Musculoskeletal Diseases (REMEDY), Diakonhjemmet Hospital, Oslo, Norway; Center for Treatment of Rheumatic and Musculoskeletal Diseases (REMEDY), Diakonhjemmet Hospital, Oslo, Norway; Center for Treatment of Rheumatic and Musculoskeletal Diseases (REMEDY), Diakonhjemmet Hospital, Oslo, Norway; Institute of Clinical Medicine, University of Oslo, Oslo, Norway; Center for Treatment of Rheumatic and Musculoskeletal Diseases (REMEDY), Diakonhjemmet Hospital, Oslo, Norway; Institute of Clinical Medicine, University of Oslo, Oslo, Norway; Center for Treatment of Rheumatic and Musculoskeletal Diseases (REMEDY), Diakonhjemmet Hospital, Oslo, Norway; Public Health Section, Inland Norway University of Applied Sciences, Elverum, Norway

**Keywords:** serious infection, bDMARD, biologics, rheumatoid arthritis, spondyloarthritis, psoriatic arthritis

## Abstract

**Objectives:**

To compare the risk of serious infection across time cohorts in patients with inflammatory arthritis (IA) initiating their first biologic/targeted synthetic DMARD (b/tsDMARD), to that of the general population. Secondarily, to compare the development in infection risk during treatment across diagnoses and examine risk dynamics during the course of b/tsDMARD treatment.

**Methods:**

Patients with IA starting their first b/tsDMARD were included from the prospective NOR-DMARD study. Controls were randomly drawn from the general population. Cox regressions were used to compare the 12-month risk of serious infections across three time cohorts following initiation (2009–2011, 2012–2014, 2015–2018) and risk during the course of treatment at 6-month intervals up to 24 months.

**Results:**

A total of 4309 patients (RA, 1581; PsA, 1032; SpA, 1696) and 86 640 controls were included. From 2009 through 2018, 51 serious infections occurred during the first year of b/tsDMARD treatment in RA patients [hazard ratio (HR) 2.42 (95% CI 1.83, 3.21)] compared with controls and 52 serious infections were observed in patients with PsA/SpA [HR 1.91 (95% CI 1.44, 2.52)]. There were no significant differences in 12-month risk of serious infections during b/tsDMARD exposure between time cohorts. PsA/SpA patients had a consistently lower risk of serious infection compared with RA patients. The risk of serious infections did not change during the treatment course.

**Conclusion:**

Patients with IA starting their first b/tsDMARD between 2009 and 2018 had a consistently higher 12-month risk of serious infection compared with controls. No change in the risk of serious infection across time cohorts of b/tsDMARD initiation was observed, nor during the treatment course.

Key messages

- Patients with inflammatory arthritis on b/tsDMARD therapy had a consistently higher risk of serious infections than the general population.
- The 12-month risk of serious infection after initiating b/tsDMARD was similar across time cohorts in both patient groups.
- No changes in infection risk were observed during the course of b/tsDMARD treatment for either patient group.

## Introduction

Patients with inflammatory arthritis (IA) have an increased risk of serious infection compared with the general population [1–4]. However, the bulk of studies comparing the risk between IA patients and the general population have been conducted before biologic and targeted synthetic DMARDs (b/tsDMARDs) were used extensively and have primarily focused on patients with RA, with less data available for PsA and SpA.

The increased susceptibility to serious infections in IA patients is believed to be multifactorial, caused by both intrinsic factors such as immunological dysfunction related to the disease process, disease-associated comorbidities and functional disability, and extrinsic factors such as immunological dysfunction due to the use of immunosuppressive therapies [5, 6]. High disease activity may lead to the need for concomitant glucocorticoid treatment with additional enhanced immunosuppression and thus increased susceptibility for infections.

Over the last two decades, treatment strategies have changed considerably alongside the introduction and approval of several novel b/tsDMARDs. Current treatment recommendations include early treatment initiation aiming for sustained remission or low disease activity using a treat-to-target approach [7–13]. Considering that high disease activity is associated with an increased risk of serious infections [6], the risk of serious infection could be expected to become attenuated in these patients due to more effective treatment, resulting in preserved functional levels and reducing the need of glucocorticoids. However, the reduction in risk may be counterbalanced by the increased level of immunosuppression due to b/tsDMARD use.

Little is known about how improvements in treatment and treatment strategies have affected the risk over the last two decades for the larger IA population, despite changes in drug exposure and treatment strategies [4, 14–16]. Data on the comparative risk of serious infection in IA patients and the general population in the biologic era is particularly scarce for patients with PsA and SpA [2, 3, 17], as the majority of previous studies addressing this issue have focused on RA patients [2, 4, 16, 18, 19].

Treatment with b/tsDMARDs has been associated with an increased risk of serious infections [20–22]. The infection risk related to b/tsDMARD exposure appears to change over the course of treatment, where several studies on RA patients have reported a time-dependent risk during treatment with TNF inhibitors (TNFis) or non-TNFi bDMARDs, with the highest risk during the first 6–12 months of treatment [20, 21, 23–26]. However, the majority of previous studies on infection risk during the course of b/tsDMARD treatment have focused on RA [23–26], with only few observational studies exploring the risk of serious infections in patients with SpA or PsA treated with biologics [27–30]. Identifying how these immune-modulating drugs affect the susceptibility to infection is still of great importance both when initiating b/tsDMARD treatment and during follow-up. Knowledge is also needed regarding if and how the risk of serious infections changes during the course of treatment with a b/tsDMARD in the larger IA population.

The objectives of this study were 3-fold: to compare the 12-month risk of serious infections in patients with RA, PsA and SpA starting their first b/tsDMARD with population controls across successive periods of treatment initiation; to compare the risk across time periods of treatment initiation within each patient population and across diagnoses; and to explore if the risk of serious infection changes during the course of b/tsDMARD treatment in 6-month intervals.

## Methods

### Data sources

In the present study, data from the ongoing NORwegian Disease-Modifying AntiRheumatic Drug (NOR-DMARD; NCT01581294) register were used. NOR-DMARD is a prospective, observational study established in 2000 with the objective of examining the long-term safety and effectiveness of bDMARD treatment (later expanded with tsDMARDs) in a real-life setting [31]. Adult patients (≥18 years) with an IA who start or switch b/tsDMARD treatment at one of the participating rheumatology centres are eligible for enrolment in the NOR-DMARD. The number of recruiting centres decreased during the study period. Clinical diagnoses are made by a rheumatologist and supported by international classification criteria.

Data collected at each study visit include disease activity assessments, patient-reported outcome measures and adverse events. Blood samples are analysed for markers of inflammation. A standardized disease activity measure based on the disease activity measurement specific for each diagnosis was used to capture disease activity for all three IA diseases. For RA, the 28-joint Disease Activity Score with CRP (DAS28-CRP) [32] was used, for SpA the Ankylosing Spondylitis Disease Activity Score (ASDAS) was used [33, 34] and for PsA the 28-joint Disease Activity in PSoriatic Arthritis (DAPSA28) score was used [33]. For PsA patients with missing information on DAPSA28, the DAS28-CRP was used when calculating disease activity. Study visits are performed at pre-specified time points after treatment start. Inclusion and exclusion criteria for the NOR-DMARD study are described in the Supplementary Data S1, available at *Rheumatology Advances in Practice* online.

The NOR-DMARD study was granted ethical approval by the Regional Committees for Medical and Health Research Ethics South East Norway (reference number: REK 2011/1339). Linkage of the NOR-DMARD study to relevant national registries and to the general population through Statistics Norway was approved by the Regional Committees for Medical and Health Research Ethics South East Norway (reference numbers: REK 2017/2041 and REK 2017/243). To be included in the NOR-DMARD study, all patients have to provide written informed consent.

### Study population

The present study included bio-naïve patients participating in the NOR-DMARD study diagnosed with RA, PsA or SpA and starting treatment with their first b/tsDMARD between January 2009 and March 2020 (Supplementary Fig. S1, available at *Rheumatology Advances in Practice* online). To secure a sufficient number of events in the analyses, PsA and SpA patients were grouped together in this study. This choice is supported by the Assessment of SpondyloArthritis international Society definition of PsA as an entity within the spectrum of SpA [35–37]. Immunosuppressive medications were categorized into the following categories: conventional synthetic DMARDs, TNFi monotherapy, TNFi combination therapy, interleukin inhibitors, abatacept, Janus kinase inhibitors and rituximab.

### General population comparator cohort

General population subjects constituted the control population. For each patient in the NOR-DMARD, 10 controls, matched on age, sex and residential area, were randomly selected by Statistics Norway from the general population and the personal identification numbers were removed to ensure anonymity. Each control was given an index date equal to the date of b/tsDMARD initiation of the corresponding patient and followed prospectively. Included in this current study are IA patients who received a first b/tsDMARD, while all comparators with an index date between 2009 and March 2020 were included, which explains the high number of controls.

### Follow-up

Patients were followed from inclusion in the NOR-DMARD study at the time of initiation of the first b/tsDMARD treatment until the first occurrence of the following: first serious infection, discontinuation of first b/tsDMARD treatment, withdrawal from NOR-DMARD, death, emigration or censor date (31 January 2019–1 March 2020). To explore if the incidence and risk of serious infection changed across successive periods of b/tsDMARD initiation, the study population was divided into three time cohorts according to the year of treatment initiation with the first b/tsDMARD: 2009–2011, 2012–2014 or 2015–2018. The censor date was set as 31 December 2018 in the third time cohort to ensure a maximum follow-up time of 12 months. When exploring the risk during a treatment course, patients starting treatment up to 1 March 2020 were included. When comparing risk against population controls, only data from the first 12 months after treatment initiation were used. When exploring the risk within the course of treatment, data up to 24 months after treatment initiation were used. We considered patients discontinuing treatment to still be under exposure 30 days after drug discontinuation.

### Register linkages

All patients and controls were linked to the Norwegian Patient Registry (NPR) and Norwegian Cause of Death Registry (NCDR) to identify events of serious infection [for a list of International Classification of Diseases, Tenth Revision (ICD-10) codes of included diagnoses, see Supplementary Table S1, available at *Rheumatology Advances in Practice* online]. Register linkages were approved by the Regional Committees for Medical and Health Research Ethics South East Norway (REK 2017/2041, REK 2017/243).

#### NPR

Data on comorbidities (chronic obstructive pulmonary disease and/or asthma, chronic renal disease, diabetes, inflammatory bowel disease, myocardial infarction and malignancy) were retrieved from the NPR. Register linkage was performed using unique personal identification numbers. Diagnoses at discharge from hospital stays or related to other contacts with specialist healthcare services without hospital admissions (e.g. outpatient clinics) are reported to the NPR by the attending physician according to the ICD. Reporting to NPR was made mandatory in 2008 and is done using a personal identification number. A change in reporting starting on 1 January 2009 led to more complete data, and this date was thus chosen as the first time point of inclusion in the current study [38].

#### NCDR

The NCDR contains information of primary and contributory causes of death. Causes of death are classified in accordance with the ICD-10 and reported to the NCDR by the physician.

### Outcomes

The outcome of this study was serious infection. A serious infection was defined as the first infection requiring hospital admission with one or more overnight stays and/or as an infection causing death according to a predefined list of ICD-10 diagnoses during the entire study period (Supplementary Table S1, available at *Rheumatology Advances in Practice* online). To fulfil the defined criteria of serious infection, the infection had to be listed as the main discharge diagnosis or as the first contributory discharge diagnosis if the main discharge diagnosis was RA, PsA or SpA. Only the first serious infection after treatment initiation per individual was included in the analyses.

### Statistical analyses

Baseline characteristics are presented as mean (s.d.), median [interquartile range (IQR)] or frequency (%) as appropriate. Crude incidence rates (IRs) of serious infections were calculated per 100 person-years with the 95% CI for each diagnosis group and comparators. Directed acyclic graphs (DAGs) were constructed to explore how factors might influence the relationship between the exposure variable and the outcome variable and to identify covariates that could act as confounders [39] (Supplementary Fig. S2A and B, available at *Rheumatology Advances in Practice* online). Multicollinearity between covariates was explored in correlation matrices and the covariates included in the models were not strongly correlated. Cox regression models were applied to compare the risk of serious infection between patients and controls during the first 12 months following initiation of the first b/tsDMARD in different time cohorts and between 6-month intervals (0–6, 6–12, 12–18 and 18–24 months, with 18–24 months as reference) during the treatment course for RA patients and PsA/SpA patients.

The models comparing the risk of serious infections in patients and controls were adjusted for age and sex, and hazard ratios (HRs) were estimated. The comparison of risk across diagnoses was explored in Cox regression analyses adjusted for age, sex, standardized disease activity measurement, disease duration prior to b/tsDMARD initiation and baseline use of methotrexate and/or prednisolone. Cox regression analyses exploring the risk of serious infections during treatment course were adjusted for age, sex, diagnosis, standardized disease activity measurement, previous serious infection and baseline co-medication with methotrexate and/or prednisolone. The likelihood ratio test was utilized to explore if the HR varied across time cohorts. Missingness was assumed to be at random. All analyses were performed in Stata versions 16 and 17 (StataCorp, College Station, TX, USA) and GraphPad Prism 9.4.1 was used to create forest plots (GraphPad Software, Boston, MA, USA).

## Results

### Population characteristics

A total of 4309 patients (1581 RA, 1032 PsA and 1696 SpA) and 86 640 comparators from the general population were included in the present study. Characteristics of the study population, as well as information on missing data, are presented in Table 1 and supplementary characteristics in Supplementary Table S2 and Table S3, available at *Rheumatology Advances in Practice* online.

**Table 1.** Baseline characteristics of patients and population controls

Characteristics	All patients (*n* = 4309)	RA (*n* = 1581)	PsA (*n* = 1032)	SpA (*n* = 1696)	Controls (*n* = 86 640)
Demographics					
Age (years), mean (s.d.)	47.7 (14.0)	53.8 (14.0)	48.1 (12.3)	41.8 (12.3)	48.4 (14.2)
Female, *n* (%)	2443 (57)	1158 (73)	547 (53)	738 (44)	54 981 (63)
Disease duration, months, median (IQR)^a^^,^^b^	5.0 (1.2, 12.9)	6.3 (2.1, 14.3)	5.2 (1.3, 11.4)	3.2 (0.6, 12.8)	–
DAS28-CRP, mean (s.d.)^c^	–	4.0 (1.3)	3.5 (0.9)	–	–
DAPSA28, median (IQR)^c^	–	–	16.9 (12, 25)	–	–
ASDAS, mean (s.d.)^c^	–	–	–	2.95 (0.96)	–
CRP, median (IQR)^c^	5 (2, 12)	5 (2, 14)	4 (2, 10)	5 (2, 11)	–
MHAQ, median (IQR)^c^	0.50 (0.25, 0.88)	0.57 (0.25, 1)	0.63 (0.25, 0.88)	0.50 (0.25, 0.88)	–
Patient global assessment, median (IQR)	36 (15, 62)	34 (12, 61)	39 (18, 63)	35 (15, 63)	
Comorbidities, *n* (%)					
COPD and/or asthma	35 (0.8)	17 (1.1)	7 (0.7)	11 (0.7)	1136 (1.3)
Chronic renal disease	11 (0.3)	6 (0.4)	3 (0.3)	2 (0.1)	262 (0.3)
Diabetes	49 (1.1)	21 (1.3)	21 (2.0)	7 (0.4)	1693 (2.0)
Inflammatory bowel disease	45 (1.0)	6 (0.4)	5 (0.5)	34 (2.0)	387 (0.5)
Myocardial infarction	7 (0.2)	2 (0.1)	2 (0.2)	3 (0.2)	288 (0.3)
Malignancy	52 (1.2)	24 (1.5)	11 (1.1)	17 (1.0)	3302 (3.8)
Previous serious infection^d^	377 (8.8)	170 (10.8)	79 (7.7)	128 (7.6)	4456 (5.1)
Medication, *n* (% of total number patients)					
TNFis					–
Monotherapy	1551 (36)	105 (7)	251 (24)	1195 (71)	
Combination therapy^e^	2432 (56)	1205 (76)	749 (73)	478 (28)	
Non-TNFi bDMARD^f^	326 (8)	271 (17)	32 (3)	23 (1)	
Co-medication, *n* (% of total number patients)					
Prednisolone^g^^,^^h^	1087 (25)	737 (47)	189 (18)	99 (6)	–
<7.5 mg	526 (12)	380 (24)	106 (10)	40 (2)	
7.5 –14 mg	321 (7)	245 (15)	49 (5)	27 (2)	
≥15 mg	178 (4)	112 (7)	34 (3)	32 (2)	
Methotrexate	1887 (44)	1110 (70)	617 (60)	160 (9)	–
Other DMARDs^i^	715 (16)	433 (27)	175 (16.5)	110 (6.3)	–

MHAQ: Modified Health Assessment Questionnaire; COPD: chronic obstructive pulmonary disease.

There were no missing data on age and sex.

a Disease duration before start of first b/tsDMARD.

b Missing disease duration: RA, 348; PsA, 267; SpA, 581.

c Missing DAS28-CRP: RA = 152, PsA = 104; missing DAPSA28: PsA = 460; missing ASDAS: SpA = 307; missing CRP: RA = 58, PsA = 36, SpA = 56; missing MHAQ: RA = 81, PsA = 51, SpA = 75; missing PGA: RA = 11, PsA = 66, SpA = 130.

d No data prior to 2009.

e TNFi in combination with a DMARD and/or prednisolone.

f Non-TNFi bDMARDs: rituximab, interleukin inhibitors (tocilizumab, iksekizumab, ustekinumab, secukinumab), abatacept, Janus kinase inhibitors (tofacitinib, baricitinib).

g Mean dose of prednisolone: RA, 7 mg (s.d. 6); PsA, 6 mg (s.d. 7); SpA 4 mg (s.d. 8).

h Missing prednisolone dose in prednisolone users: RA, 30/767; PsA, 18/207; SpA, 14/113.

i Other DMARDs: leflunomide, sulfasalazine and hydroxychloroquine.

### Risk of serious infections following b/tsDMARD initiation in IA patients *vs* the general population

Among patients starting their first b/tsDMARD between year 2009 and 2019, a total of 51 serious infections occurred within the first 12 months after treatment initiation in 1479 RA patients (12-month IR 4.45) and 52 serious infections were observed in 2544 PsA/SpA patients (12-month IR 2.59), with an IR of 1.54 in the general population (Table 2). The risk of serious infections was significantly higher in patients compared with the general population in those initiating b/tsDMARD therapy between 2009 and 2018, with an HR of 2.24 (95% CI 1.83, 3.21) in RA patients *vs* controls and HR 1.91 (95% CI 1.44, 2.52) in PsA/SpA patients *vs* controls (Table 2).

**Table 2.** Risk of serious infection during first 12 months of treatment with first b/tsDMARD initiated between 2009 and2018

Variables	RA	PsA/SpA	Controls
Patients, *n*	1479	2544	80 427
Events, *n*	51	52	1231
Person-years	1145	2007	79 688
IR per 100 person-years (95% CI)	4.45 (3.38, 5.86)	2.59 (1.97, 3.40)	1.54 (1.46, 1.63)
HR^a^ (95% CI)	2.42 (1.83, 3.21), *P* < 0.001	1.91 (1.44, 2.52), *P* < 0.001	Ref.
HR^a^ (95% CI)	Ref.	0.71 (0.46, 1.09), *P* = 0.120	
HR^b^ (95% CI)	Ref.	0.52 (0.28, 0.95), *P* < 0.05	

Missing standardized disease activity measurement: RA, 125/1479; SpA, 276/1568; PsA, 91/976 (DAS28-CRP used as disease activity measure for PsA patients with missing information on DAPSA28). There were no missing data on age and sex.

a Adjusted for age and sex.

b Adjusted for age, sex, standardized disease activity measurement, disease duration, baseline co-medication and use of methotrexate and/or prednisolone.

### The comparative risk of serious infection between IA patients and the general population over time

To explore if the comparative risk of serious infection has changed over time between IA patients and the general population, we assessed the risk in time cohorts based on the year of treatment initiation, as described in the Methods section. The risk of serious infections was consistently higher in patients compared with controls in all three time cohorts for both RA and PsA/SpA patients (Table 3). The HR of serious infections in RA patients compared with population comparators numerically declined slightly over time (from HR 2.79 in cohort 1 to HR 2.18 in cohort 3), however, this decline in the comparative risk across time cohorts was not statistically significant. Few events in the combined group of PsA/SpA patients restricted the comparison of the risk to that of the general population over time (Table 3).

**Table 3.** Risk of serious infection during first 12 months of treatment with first b/tsDMARD after time cohort (years)

Variables	Cohort 1 (2009–2011)	Cohort 2 (2012–2014)	Cohort 3 (2015–2018)
RA			
Patients, *n*	582	435	462
Events, *n*	23	14	14
Person-years	453	334	357
IR per 100 person-years (95% CI)	5.07 (3.37, 7.63)	4.19 (2.48, 7.07)	3.92 (2.32, 6.62)
HR^a^ (95% CI)	Ref.	0.79 (0.40, 1.53)	0.72 (0.37, 1.41)
HR^b^ (95% CI)	Ref.	1.03 (0.50, 2.14)	1.13 (0.53, 2.39)
PsA/SpA			
Patients, *n*	772	902	870
Events, *n*	15	15	22
Person-years	617	720	670
IR per 100 person-years (95% CI)	2.43 (1.46, 4.03)	2.08 (1.26, 3.46)	3.28 (2.16, 4.99)
HR^a^ (95% CI)	Ref.	0.84 (0.41, 1.73)	1.30 (0.67, 2.51)
HR^b^ (95% CI)	Ref.	0.45 (0.15, 1.38)	1.44 (0.60, 3.45)
Controls			
Patients, *n*	31 653	23 542	25 232
Events, *n*	502	358	371
Person-years	31 344	23 333	25 011
IR per 100 person-years (95% CI)	1.60 (1.47, 1.75)	1.53 (1.38, 1.70)	1.48 (1.34, 1.64)
HR^a^ (95% CI)	Ref.	1.00 (0.87, 1.15)	0.95 (0.83, 1.08)
Patients *vs* controls			
HR^a^ (95% CI)	2.79 (1.83, 4.23), *P* < 0.001	2.20 (1.29, 3.76), *P* < 0.05	2.18 (1.27, 3.72), *P* < 0.05
RA *vs* controls (ref.)			
HR^a^ (95% CI)	1.84 (1.10, 3.09), *P* = 0.021	1.50 (0.89, 2.52), *P* = 0.124	2.42 (1.57, 3.73), *P* < 0.001
PsA/SpA *vs* controls (ref.)			

Missing standardized disease activity measurement: RA, 125/1479; PsA, 91/976 [DAS28-CRP used as disease activity measure for PsA patients with missing information on DAPSA28 (missing DAPSA28, 446/976)]; SpA, 276/1568.

a Adjusted for age and sex.

b Adjusted for age, sex, standardized disease activity measurement, disease duration and baseline co-medication with methotrexate and/or prednisolone.

### Comparison of the risk of serious infections across time cohorts and diagnoses

**Figure 1.**
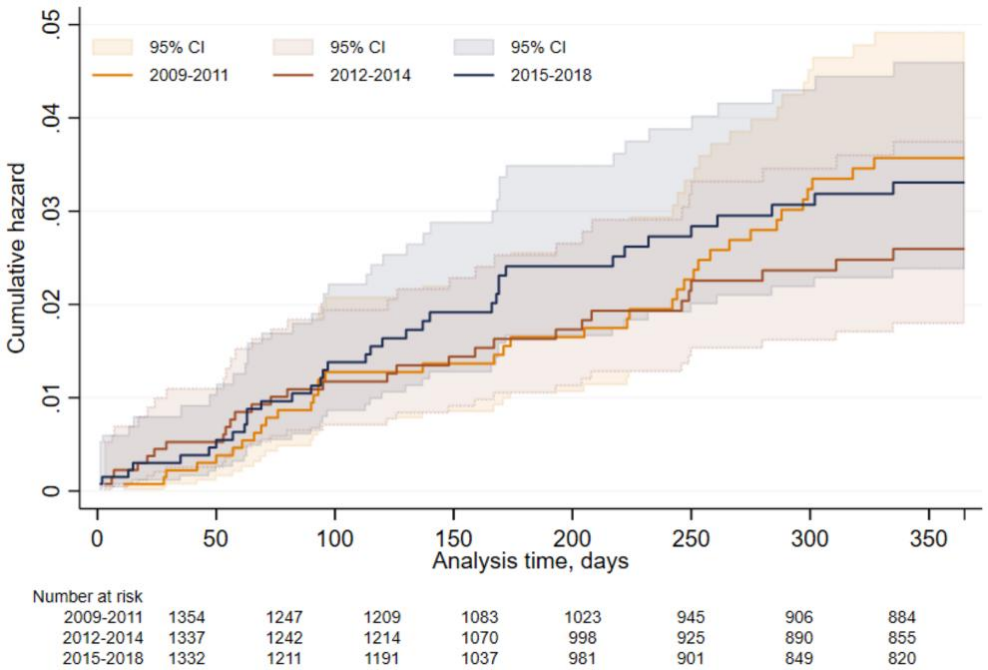
Cumulative hazard of first serious infection within first 12 months of treatment with first b/tsDMARD across time cohorts according to year of treatment initiation. Nelson–Aalen plot of 12-month cumulative hazards with 95% CIs across three cohorts according to year of treatment initiation; 2009–2011, 2012–2014 and 2015–2018

We did not find a significant change in the risk of serious infection for either RA or PsA/SpA patients across time cohorts of b/tsDMARD initiation year with cohort 1 (2009–2012) as the reference (Fig. 1, Table 3). The risk of serious infection during the first year of b/tsDMARD therapy initiated between 2009 and 2018 was significantly lower in PsA/SpA patients compared with RA patients in adjusted analysis with an adjusted HR (adjHR) of 0.52 (95% CI 0.28, 0.95).

### The risk of serious infections during treatment course with a b/tsDMARD

**Figure 2.**
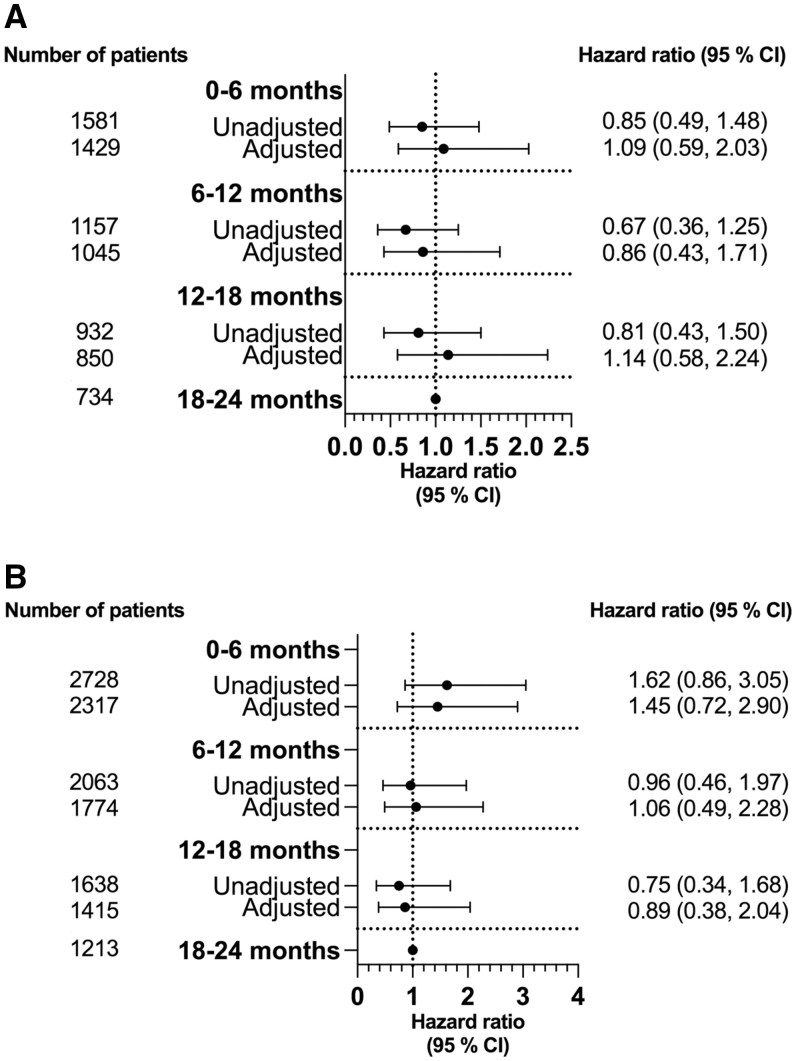
Risk of serious infection during treatment course with first b/tsDMARD in patients with **(A)** RA and **(B)** PsA/SpA. Reference was 18–24 months of treatment. Adjustments for age and sex in unadjusted Cox regression analyses. In the adjusted Cox regression analyses, we adjusted for age, sex, diagnosis, standardized disease activity measurement, previous serious infection and baseline co-medication with methotrexate and/or prednisolone. In PsA patients, the DAS28-CRP was used as the disease activity measure if DAPSA28 was missing

During the first 24 months of treatment with the first b/tsDMARD, a total of 91 and 79 serious infections occurred in RA and PsA/SpA patients, respectively. The risk was similar during the treatment course for either diagnosis group, with an adjHR for 0–6 months *vs* 18–24 months of treatment of 1.25 (95% CI 0.65, 2.42) in RA patients and an adjHR of 1.13 (95% CI 0.53, 2.44) in PsA/SpA patients (Fig. 2). We found that the risk of serious infections was consistently lower during the treatment course in PsA/SpA *vs* RA patients, but this did not reach statistical significance (Supplementary Table S4, available at *Rheumatology Advances in Practice* online).

## Discussion

This large, register-linked study explored the risk of serious infection in patients with RA, PsA or SpA during b/tsDMARD exposure compared with matched population controls. Our study provides insights on the rate and risk of serious infection over time not only in patients with RA, but also in those with PsA and SpA starting their first b/tsDMARD. We found a nearly 2-fold higher 12-month risk of serious infections in patients compared with controls. Compared with controls, the 12-month risk of serious infections was consistently higher in patients initiating a b/tsDMARD according to the treat-to-target principles in all three time cohorts. These results indicate that serious infections are also of importance in modern treatment of IA patients. The risk of serious infection did not change significantly over time in either of the two diagnosis groups, nor during the course of treatment.

Our findings of a consistently higher risk in patients compared with population controls is in line with results from other studies comparing the risk of serious infections in IA patients with the general population, both in the pre-biologic and in the biologic era [2, 4, 19, 40]. In the pre-biologic era, RA patients were reported to have twice the risk of serious infections compared with the general population [19], while a recent comparison of the risk of serious infections in patients with IA starting their first bDMARD found the risk to be four times higher in IA patients compared with the general population [2]. The discrepancy in risk of serious infections between our study and that reported in the Danish cohort study could be due to differences in age, disease activity and follow-up time.

We did not find a statistically significant reduction in the risk of serious infections in successive treatment cohorts compared with the general population in either RA or PsA/SpA patients. A decline in risk over time in bio-naïve RA patients starting b/tsDMARD treatment compared with the general population could have been expected due to improved disease activity following the modern treat-to-target approach. In a study by Zhou *et al.* [4], the risk of serious infection was found to be increased in RA patients diagnosed in the biologic era compared with a cohort of RA patients diagnosed before the introduction of biologics, and the difference in rates of serious infections increased in RA patients and non-RA controls after bDMARD introduction. However, the cohorts were heterogeneous and not all patients were treated with bDMARDs in the biologic era cohort, thus the results are not directly comparable to the results in this current study.

We did not find a significant change in the risk of serious infections when the risk was explored within each diagnosis group across time cohorts for RA or PsA/SpA patients. Despite changes in treatment strategies since the implementation of bDMARDs, few studies have examined the risk of serious infections in successive cohorts of patients while on b/tsDMARDs, and again pertains to RA. In a study comparing patients diagnosed with RA between 1955 to 1994 with those diagnosed between 1995 and 2007, a decline in the overall rate of serious infections was reported, but again the treatments were heterogeneous; very few patients were treated with bDMARDs in the early cohort and only 29% in the later cohort [14]. Similarly, a recent study using data from Brazilian and Argentinian registries reported a decrease in IRs of serious infection from 2010 to 2016 in RA patients treated with bDMARDs [15]. In addition, a Japanese study exploring the risk of serious infections over time in 1068 RA patients divided into two time cohorts depending on the year of TNFi initiation also found a significant decreased risk of serious infections across successive cohorts [16]. However, these studies included only RA patients and not other IA diseases.

While the risk of serious infection during the treatment course has been less described in PsA/SpA patients, previous studies have addressed this issue in RA patients and report a higher risk of serious infections within the first 6 months of treatment with a bDMARD [15, 21, 23–26]. In contrast, we did not find a statistically significant change in the risk of serious infections during b/tsDMARD treatment courses for RA or PsA/SpA patients. The lack of a statistically significant trend of reduction in the risk of serious infections during the treatment course in the current study could be due to the small number of events. and thus a lack of power, or that the risk is not time dependent as indicated in previous studies [15, 21, 23–26].

This study has some limitations. First, data on glucocorticoid use were suboptimal, as only data on baseline use were valid. Further, information on shorter glucocorticoid exposures during the first year of DMARD treatment were not available. Their potential to increase the risk of serious infections is known to be duration-dependent as well as dose-dependent, and the lack of valid information on initiation or discontinuation of glucocorticoids is an important limitation to be considered. Not all b/tsDMARDs are approved for use in PsA/SpA patients, causing differences in the distribution of the different b/tsDMARDs between RA and PsA/SpA patients. Some of the treatments, e.g. rituximab, are exclusively used in RA, with a higher infection risk due to their mode of action, possibly explaining, at least to some degree, the observed difference in the risk of serious infections across diagnoses. However, the percentage of RA patients treated with rituximab (8%) and abatacept (3%) was low, thus reducing the impact of these differences on the results. Further, channelling bias applies when patients with a higher susceptibility of serious infection are treated with non-TNFi bDMARDs, leading to a poorer safety profile in these patients, and needs to be considered when interpreting the results. Further, missing data on variables could have an impact on infection risk, such as smoking and change in disease activity during treatment. Finally, the small number of events limits the statistical power in our study and the treatment period was defined as up until the end of treatment plus 30 days for all individuals, regardless of pharmacokinetics and individual variations in serum drug concentrations.

One of the main strengths of this study is the prospective study design with multicentre register data reflective of clinical practice in which participating patients are treated according to the current recommendations. Further strengths include the use of data from a large cohort of patients with different IA diseases followed regularly in a real-life setting and matching to comparators from the general population. Additionally, the use of high-quality data from national registries with high completeness and validated data on diagnoses related to contacts with specialist healthcare services or death according to the ICD-10 ensured accurate capture of the outcome. The definition of the outcome variable, serious infections, is identical to that used in other studies, adding to the validity of the outcome.

In conclusion, the 12-month risk of serious infection in patients starting b/tsDMARD treatment for IA was approximately two times higher in bio-naïve IA than in matched controls. These results reaffirm the elevated risk of serious infections in patients with IA compared with the general population and highlight that clinicians need to be aware of serious infections during the course of treatment.

## Supplementary Material

rkaf017_Supplementary_Data

## Data Availability

Data will be made available upon reasonable request.
