# Supplementary material for: Longitudinal risk of serious infections in patients with inflammatory arthritis on immunomodulating therapy compared to controls
Source: Rheumatol Adv Pract. 2025 Feb 12;9(1):rkaf017. doi: 10.1093/rap/rkaf017 (PMC11889454; doi:10.1093/rap/rkaf017)
Supplement: rkaf017_Supplementary_Data [file rkaf017_supplementary_data.zip › 24-072 Supplementary Material.docx]

**Supplementary material**

I E Christensen et al. **Longitudinal risk of serious infections in patients with inflammatory arthritis on immunomodulating therapy compared to controls**

Table of Contents

[Section 1. Inclusion and Exclusion Criteria 2](#_Toc159875432)

[Section 2. ICD-10 codes 3](#_Toc159875433)

[Table S1 International Classification of Disease version 10 (ICD-10) codes 3](#_Toc159875434)

[Section 3. Supplementary figures 4](#_Toc159875435)

[Section 4. Supplementary tables 6](#_Toc159875436)

[Table S2 Supplementary baseline characteristics 6](#_Toc159875437)

[Table S3 Baseline characteristics across time-cohorts after diagnoses 7](#_Toc159875438)

# Section 1. Inclusion and Exclusion Criteria

**Data S1** Inclusion and Exclusion Criteria

|  | |
| --- | --- |
| **Inclusion Criteria** | - Diagnosis of inflammatory arthritis - Adult patients (> 18 years) - Starting treatment with a biological or targeted synthetic disease-modifying antirheumatic drug (DMARD) (up to 2012, all DMARDs) |
| **Exclusion Criterion** | - Not able or willing to provide written informed consent - Psychiatric or mental disorders, substance abuse, language barriers or other reasons making it impossible to adhere to study protocol - Participation in RCTs or other studies that are not compatible with participation in the NOR-DMARD study |

| **Medication group** |  |
| --- | --- |
| Tumor necrosis factor inhibitor | Infliximab, etanercept, golimumab, adalimumab, certolizumab pegol |
| Tumor necrosis factor inhibitor in combination | + methotrexate, sulfasalazine, hydrocychloroquine, leflunomide and/or prednisolone |
| Janus kinase inhibitor | Tofacitinib, baricitinib |
| Interleukin inhibitors | Tocilizumab, iksekizumab, ustekinumab, secukinumab |
| Abatacept |  |
| Rituximab |  |

# Section 2. ICD-10 codes

### Table S1 International Classification of Disease version 10 (ICD-10) codes

| **Comorbidities** | | |
| --- | --- | --- |
|  | **Data source** | **ICD-10** |
| Chronic obstructive pulmonary disease and/or asthma | NPR | J41-J45, J47, J84.1, J84.8, J84.9 |
| Chronic kidney disease | NPR | I12, I13, N00-N05, N07, N11, N18-N19, Q61 |
| Diabetes | NPR | E10-E14 |
| Inflammatory bowel disease | NPR | K50, K51 |
| Heart disease | NPR | I20.0, I21, I22, I23, I24, I50 |
| Malignancy | NPR | C00-C043, C45-C96 |
| **Serious infection** | | |
| **Infection site** | **Data source** | **ICD-10** |
| **Respiratory tract** | NPR and NCDR | A150-A188, A310, A370-A379, B371, B440-B450, B460, J00, J010-J22, J387, J440, J690, J80, J850-J869, J90 |
| **Central nervous system** |  | A321, A390, A812, A840, A841, A849, A85-A879, A89, G000-G028, G038-G052, G060-G08, G374 |
| **Genitourinary** |  | N10-N12, N136, N151, N159, N160, N300, N301, N308, N309, N330, N340, N370, N390, N412-N419, N510-N518 |
| **Skin/soft tissue** |  | A46, H050, K610-K614, L00, L010, L011, L020-L080, L088, L089, L303, M600, N481, N482, N499, N61, N72, N751 |
| **Bone/joint** |  | M000-M018, M462, M463, M465, M600, M630-M632, M650, M651, M680, M710, M711, M726, M728, M860-M869, K102 |
| **Sepsis** |  | A327, A391-A449, A480-A499, R572, R650, R651 |
| **Gastrointestinal/intraabdominal** |  | A000-A099, B971, K352-K358, K37, K570, K572, K574, K578, K630, K650, K659, K670-K678, K750, K770, K800, K803, K804, K810, K818, K819, K830, K850, K858, K859, K870, K871, K930 |
| **Other** |  | A190-A309, A311-A320, A328, A329, A34-A369, A38, A500-A839, A848, A850-A99, B000-B052, B054-B439, B451-B459, B461-B573, B575-B99, D733, E060, E321, E350, H000, H030-H038, H162-H169, H191, H192, H440, H600, H601, H610, H620-H624, H660, H664-H671, H700, H750, I301, I320, I321, I330, I400, I410-I412, J36, J390, J391, K041, K044, K046, K047, K052, K112, K113, K122, N450, N459, N700, N709, N730, N732, N733, N735, N738, N740-N748, N751, N764, T802, T814, T826, T827, T835, T836, T845-T847, T857, T880, Z2230 |
| NPR; Norwegian Patient Register, NCDR; Norwegian Cause of Death Registry | | |

# Section 3. Supplementary figures

**Figure S1** Flow-chart of analyses population:

**
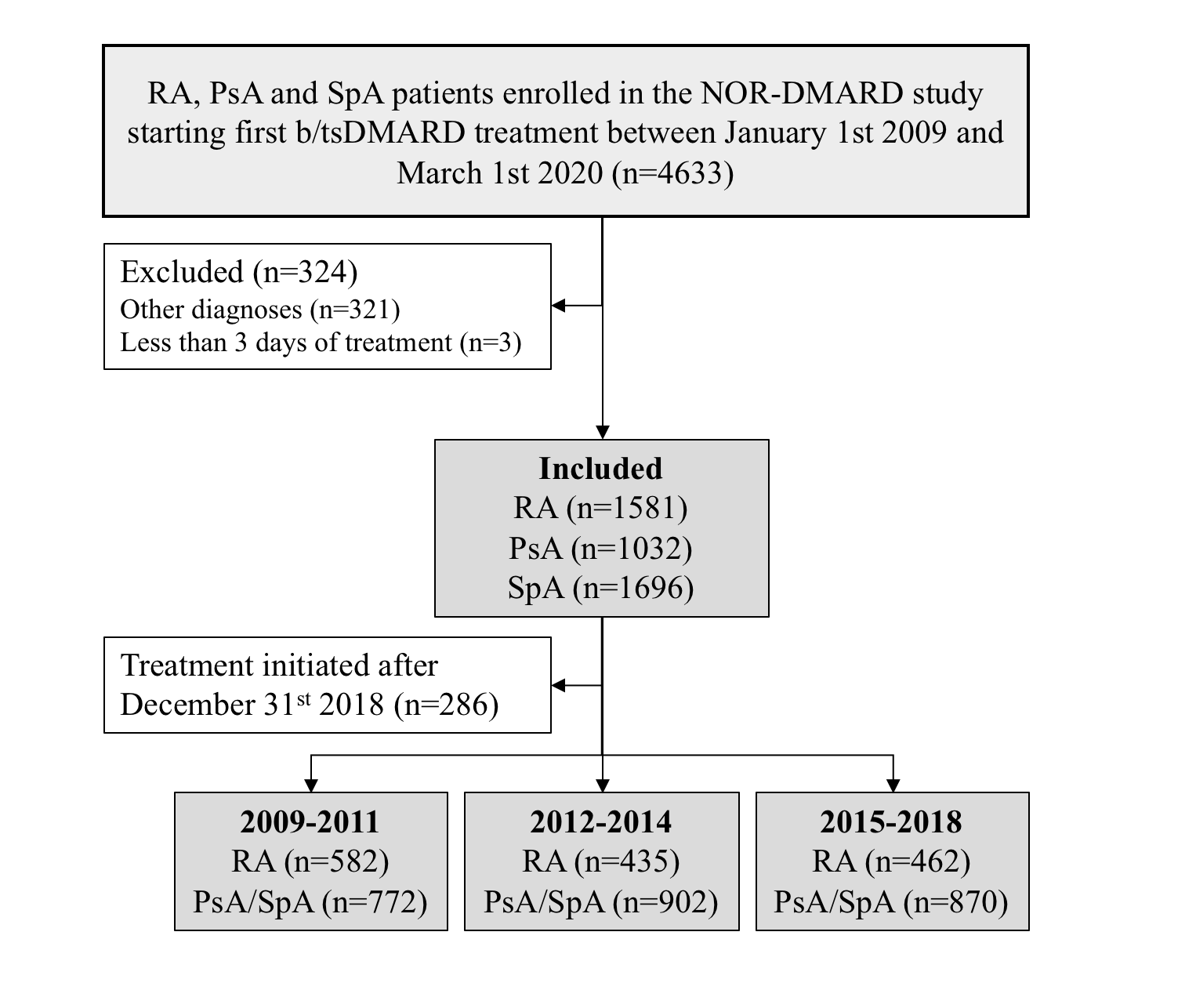
**

**Figure S1 Legend**

Other diagnoses include adult juvenile idiopathic arthritis, unspecified polyarthritis and reactive arthritis

b/tsDMARD; biological or targeted synthetic disease-modifying antirheumatic drug, RA; rheumatoid arthritis, PsA; psoriatic arthritis, SpA; spondyloarthritis

**Figure S2** Directed acyclic graphs

Dagitty were used to create directed acyclic graphs. In model a) the exposure (green) was year of start first biological or targeted synthetic DMARD. In model b) the exposure (green) was b/tsDMARD. In both models, the outcome (blue with I in center) was serious infection.

**a)** analyses on risk of serious infection across time-cohorts according to year of initiation with first biologic/targeted synthetic disease-modifying antirheumatic drug (b/tsDMARD)


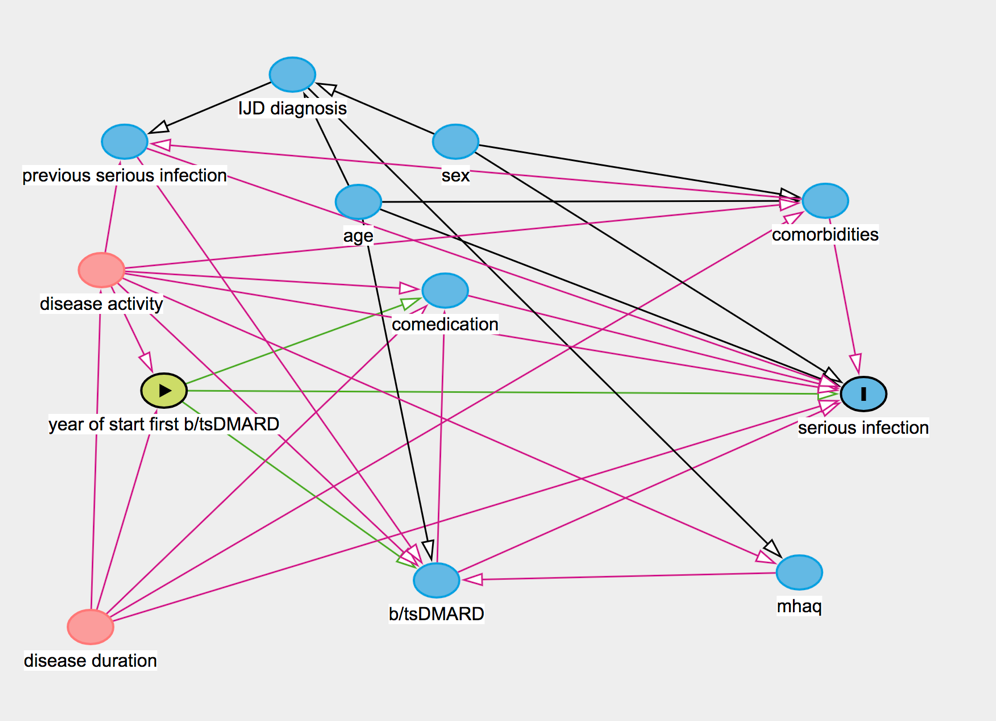


**b)** analyses on risk of serious infection during treatment course with first biologic/targeted synthetic disease-modifying antirheumatic drug (b/tsDMARD)


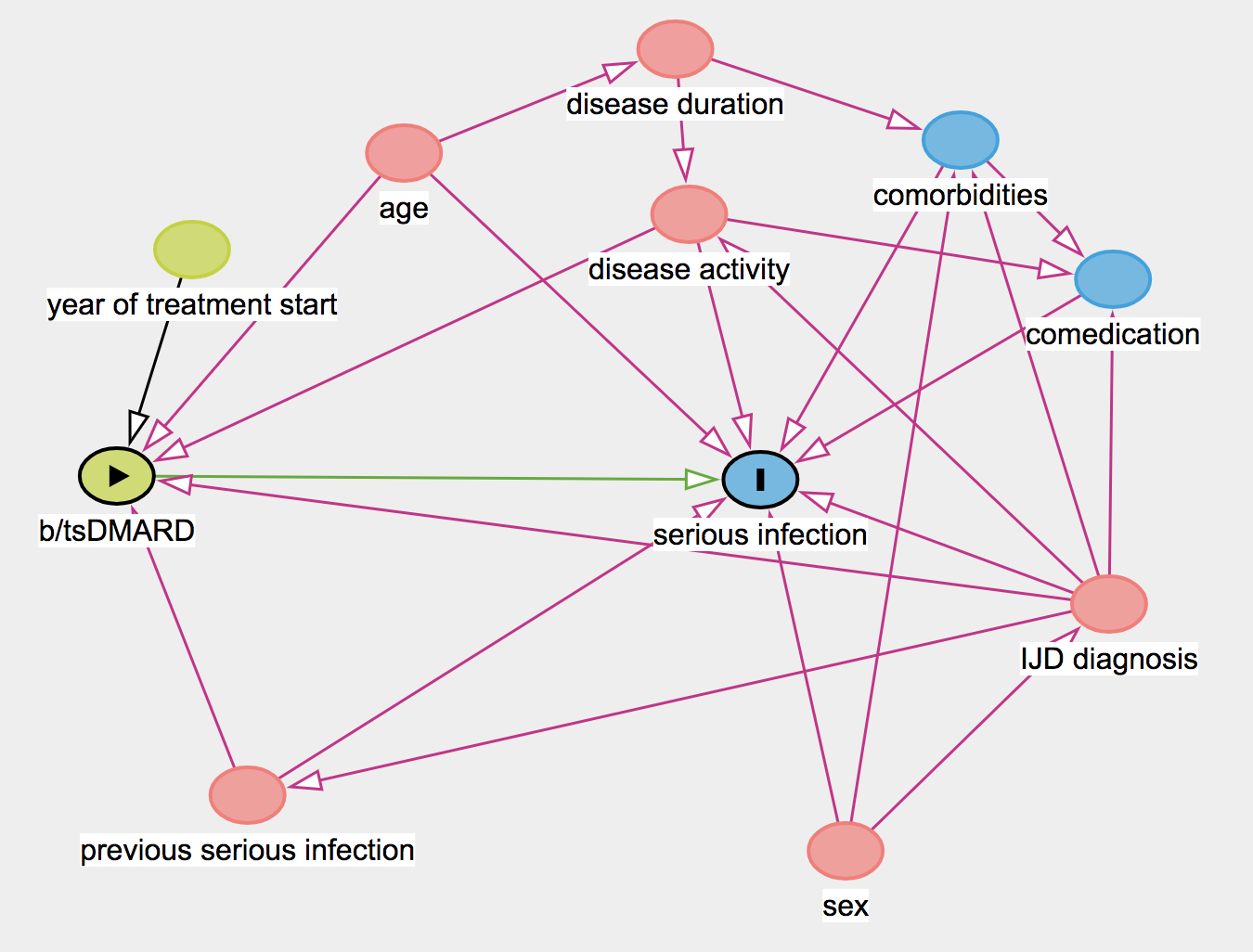


**Figure S2 Legend**

IJD; inflammatory joint disease, MHAQ; Modified Health Assessment Questionnaire, b/tsDMARD; biologic / targeted synthetic disease-modifying antirheumatic drug

# Section 4. Supplementary tables

### Table S2 Supplementary baseline characteristics

|  | **Rheumatoid arthritis**  **(n=1 581)** | **Psoriatic arthritis**  **(n=1 032)** | **Spondyloarthritis**  **(n=1 696)** |
| --- | --- | --- | --- |
| **Demographics** | | | |
| Age, n (%)  < 50 years | 539 (34) | 549 (53) | 1 217 (72) |
| ≥ 50 years | 1 042 (66) | 483 (47) | 479 (28) |
| Years on csDMARD treatment, median (IQR) | 1.8 (0.5, 5.5) | 1.9 (0.5, 5.4) | 1.8 (0.5, 5.5) |
| Disease duration, median (IQR)^§^ | 6.3 (2.1, 14.3) | 5.2 (1.3, 11.4) | 3.2 (0.6, 12.8) |
| **Medication, n (% of total number patients)** | | | |
| Rituximab | 128 (8) | 1 (0.1) |  |
| Tumor necrosis factor inhibitors |  |  |  |
| -monotherapy | 105 (7) | 251 (24) | 1 195 (71) |
| -combination therapy ^**^ | 1 205 (76) | 749 (73) | 478 (28) |
| Interleukin inhibitors | 67 (4) | 28 (2.7) | 20 (1.2) |
| Janus Kinase inhibitors | 36 (2) | 1 (0.1) | 0 |
| Abatacept | 40 (3) | 2 (0.2) | 3 (0.2) |
| nonTNFi bDMARD | 271 (17) | 32 (3) | 23 (1) |
| IQR; Interquartile range, csDMARD; conventional synthetic disease-modifying antirheumatic drug, bDMARD; biologic DMARD, TNFi; tumor necrosis factor inhibitor  Disease duration before starting with a biologic or targeted synthetic DMARD  § missing disease duration; RA= 348 PsA= 267 SpA= 581 | | | |

### Table S3 Baseline characteristics across time-cohorts after diagnoses

|  | **Cohort 1**  **2009-2011** | **Cohort 2**  **2012-2014** | **Cohort 3**  **2015-2018** |
| --- | --- | --- | --- |
| **Rheumatoid arthritis (n=1 490)** | | | |
| Age (years), mean (SD) | 53.0 (13.2) | 53.8 (14.5) | 55.0 (14.1) |
| Female, n (%) | 450 (77) | 306 (70) | 340 (72) |
| Disease duration, median (IQR)^§^ | 7.3 (2.6, 15.4) | 6.4 (2.0, 15.5) | 5.1 (1.6, 12.0) |
| DAS28-CRP, mean (SD)^§§^ | 4.3 (1.3) | 3.9 (1.2) | 3.8 (1.3) |
| CRP, mean (SD)^§§^ | 15.7 (21.8) | 10.5 (15.3) | 10.9 (16.0) |
| MHAQ, median (IQR)^§§^ | 0.63 (0.3, 1) | 0.5 (0.3, 0.9) | 0.5 (0.1, 0.9) |
| ≥ 1 Comorbidity, n (%) | 23 (4.0) | 25 (5.8) | 21 (4.4) |
| Previous serious infection, n (%) | 41 (7.9) | 38 (8.7) | 75 (15.9) |
| Tumor necrosis factor inhibitors, n (%) |  |  |  |
| -monotherapy | 13 (2.2) | 19 (4.4) | 43 (9.0) |
| -combination therapy ^**^ | 467 (80.3) | 354 (81.4) | 336 (71) |
| nonTNFi bDMARD, n (%) | 102 (17.5) | 62 (14.2) | 94 (20) |
| Prednisolone, n (%) | 323/582 (56) | 229/435 (53) | 192/473 (41) |
| < 7.5 mg | 173 | 106 | 91 |
| 7.5 mg – 14 mg | 103 | 73 | 64 |
| >= 15 mg | 25 | 43 | 36 |
| Methotrexate, n (%) | 435 (75) | 351 (81) | 284 (60) |
| **Psoriatic arthritis / spondyloarthritis (n=2 583)** | | | |
| Age (years), mean (SD) | 43.2 (12.0) | 44.5 (12.7) | 45.0 (12.7) |
| Female, n (%) | 335 (43) | 447 (50) | 438 (48) |
| Disease duration, median (IQR)^§ ¶^ | 5.2 (1.1, 13.7) | 3.0 (0.6, 10.3) | 3.9 (0.8, 11.1) |
| DAS28-CRP, mean (SD)^§§^ | 3.2 (1.1) | 3.1 (1.0) | 2.9 (1.1) |
| DAPSA28, median (IQR)^§§^ | ¨ | 14.3 (9.7, 19.3) | 13.7 (8.4, 19.6) |
| ASDAS, mean (SD)^§§^ | 3.2 (1.0) | 2.9 (0.9) | 2.7 (1.0) |
| CRP, median (IQR)^§§^ | 5 (4, 15) | 5 (2, 10) | 3 (2, 8) |
| MHAQ, median (IQR)^§§^ | 0.6 (0.3, 0.9) | 0.6 (0.3, 0.9) | 0.5 (0.3, 0.8) |
| ≥ 1 Comorbidity, n (%) | 39 (5.1) | 39 (4.3) | 41 (4.5) |
| Previous serious infection^*^, n (%) | 26 (3.4) | 67 (7.4) | 100 (11) |
| Tumor necrosis factor inhibitors, n (%) |  |  |  |
| -monotherapy | 256 (33.2) | 514 (57.0) | 571 (62.8) |
| -combination therapy ^**^ | 520 (66.0) | 385 (42.7) | 296 (32.6) |
| nonTNFi bDMARD, n (%) | 6 (0.8) | 3 (0.3) | 42 (4.6) |
| Prednisolone, n (%)^ǂ #^ | 130/772 (17) | 120/902 (13) | 63/909 (7) |
| < 7.5 mg | 53 | 58 | 30 |
| 7.5 mg – 14 mg | 29 | 29 | 17 |
| >= 15 mg | 19 | 30 | 16 |
| Methotrexate, n (%) | 215 (28) | 299 (33) | 234 (26) |
| SD; Standard deviation, IQR; Interquartile range, DAS28-CRP; Disease activity score for 28 joints, DAPSA28; Disease activity in psoriatic arthrtitis in 28 joints, ASDAS; Ankylosing spondylitis disease activity score, CRP; C-Reactive Protein, MHAQ; Modified Health Assessment Questionnaire, TNFi; tumor necrosis factor inhibitor, bDMARD; biologic disease-modifying antirheumatic drug  § missing disease duration; Rheumatoid arthritis (RA)= 348, psoriatic arthritis (PsA)= 267, spondyloarthritis (SpA)= 581  ¶disease duration before start with first b/tsDMARD  §§Missing DAS28-CRP; RA: n=152, PsA: n=104, missing DAPSA28 PsA: n=460, missing ASDAS SpA: n= 307, missing CRP: RA=58, PsA=36, SpA=56, missing MHAQ: RA=79, PsA=48, SpA=71  ^*^ no data prior to 2009  ** Tumor necrosis factor inhibitor in combination with a DMARD and/or prednisolone  ǂMissing prednisolone dose of prednisolone users: RA: 30/767, PsA: 18/207, SpA: 14/113  ^#^ mean dose prednisolone: RA=7 mg (SD 6), PsA=6 mg (SD 7), SpA=4 mg (8) | | | |

**Table S4** Risk of serious infection during treatment with first bDMARD in 6-months intervals

|  | **Rheumatoid arthritis** | **Psoriatic arthritis / spondyloarthritis** |
| --- | --- | --- |
| **0-6 months** | | |
| Number of patients, n | 1 581 | 2 728 |
| Events, n | 32 | 38 |
| Person-years | 686 | 1203 |
| IR per 100 PY | 4.67 (3.30, 6.60) | 3.16 (2.30, 4.34) |
| HR^*^, n=4 309 | Ref. | 0.83 (0.49, 1.40), p=0.479 |
| HR^**^, n=3 746 | Ref. | 0.84 (0.45, 1.58), p=0.595 |
| **6-12 months** | | |
| Number patients, n | 1 157 | 2 063 |
| Events, n | 19 | 17 |
| Person-years | 510 | 905 |
| IR per 100 PY | 3.73 (2.38, 5.84) | 1.88 (1.17, 3.02) |
| HR^*^, n=3 220 | Ref. | 0.60 (0.29, 1.25), p=0.174 |
| HR^**^, n=2 819 | Ref. | 0.62 (0.26, 1.49), p=0.289 |
| **12-18 months** | | |
| Number of patients, n | 932 | 1 638 |
| Events, n | 19 | 11 |
| Person-years | 426 | 742 |
| IR per 100 PY | 4.46 (2.84, 6.99) | 1.48 (0.82, 2.68) |
| HR^*^, n=2 570 | Ref. | 0.53 (0.23, 1.22), p=0.134 |
| HR^**^, n=2 265 | Ref. | 0.51 (0.20, 1.30), p=0.158 |
| **18-24 months** |  |  |
| Number of patients, n | 806 | 1 392 |
| Events, n | 21 | 13 |
| Person-years | 378 | 656 |
| IR per 100 PY | 5.56 (3.63, 8.53) | 1.98 (1.15, 3.41) |
| HR^*^, n=2 198 | Ref. | 0.44 (0.20, 0.97), p=0.043 |
| HR^**^, n=1 947 | Ref. | 0.58 (0.22, 1.56), p=0.280 |
| PY; person-years, HR with (95 % confidence interval); Hazard ratio, IR; Incidence Rate  ^*^basic model; adjusted for age and sex  ^**^adjusted for age, sex, diagnosis, standardized disease activity measurement, previous serious infection, baseline co-medication with methotrexate and/or prednisolone  Missing standardized disease activity measurement; RA: 418/4 476, SpA: 813/4864, PsA: 289/2957 (DAS8-CRP used as disease activity measure for PsA patients with missing information on dapsa28 (missing DAPSA28 = 446/976)) | | |
